# Comparative biomechanical analyses of lower cervical spine post anterior fusion versus intervertebral disc arthroplasty: A geometrically patient-specific poroelastic finite element investigation

**DOI:** 10.1016/j.jot.2022.05.008

**Published:** 2022-07-15

**Authors:** Kinda Khalaf, Mohammad Nikkhoo

**Affiliations:** a Department of Biomedical Engineering, Khalifa University of Science and Technology, And Health Engineering Innovation Center, Abu Dhabi, United Arab Emirates; b Department of Biomedical Engineering, Science and Research Branch, Islamic Azad University, Tehran, Iran

**Keywords:** Cervical spine, Poroelastic finite element analysis, Patient-specific modeling, Cervical anterior fusion, Disc arthroplasty

## Abstract

**Background/Objective:**

The optimal surgical technique for the treatment of cervical degenerative disc disease (CDDD) towards decreasing the risk of adjacent segment disease (ASD) remains elusive. This study aimed to comparatively investigate the biomechanics of the lower cervical spine following fusion (ACDF) and artificial disc arthroplasty (Bryan® and Prestige LP®) using a validated geometrically patient-specific poroelastic finite element modeling (FEM) approach.

**Methods:**

Ten subject-specific pre-operative models were developed and validated based on a FEM approach. Poroelastic models were then constructed using post-operation images for three different treatment scenarios: ACDF; Prestige LP® and Bryan® artificial discs at the C5–C6 level. The biomechanical responses at both surgical and adjacent spinal levels were studied subject to static and cyclic loading.

**Results:**

Postoperatively, greater range of motion (ROM), higher annulus fibrosus stress and strain values, and increased disc height and fluid loss at the adjacent levels were detected post ACDF, as compared with pre-op as well as artificial disc arthroplasty. The facet joint forces were larger for the Prestige LP® disc, particularly during extension. The lowest values in disc height and fluid exchange were observed in the Bryan® artificial disc arthroplasty models.

**Conclusion:**

Biomechanical analyses revealed that ACDF poses the highest potential risk for adjacent disc degeneration. The artificial discs investigated here (Prestige LP® and Bryan®) not only preserved motion at the instrumented level, but also sustained the pre-op ROM and decreased the intradiscal pressure (IDP) and facet joint forces (FJFs) at adjacent levels, particularly during flexion/extension. The Bryan® artificial disc demonstrated the most efficacy in maintaining the natural poroelastic characteristics of adjacent discs.

**The translational potential of this article:**

This study provided a technique for clinicians to use quantitative data towards subject-specific evaluation to comparatively evaluate the impact of ACDF and disc arthroplasty using two types of artificial discs on the biomechanics of the cervical spine. It confirms differences in the poroelastic characteristics of adjacent discs for different fixation techniques, and reveals the advantage of artificial discs with a flexible core for decreasing the risk of ASD.

## 1 Introduction

Cervical degenerative disc disease (CDDD) is one of the most common underlying pathologies of the cervical spine and a chief culprit behind an array of neurological complications, including cervical radiculopathy and myelopathy. As one of the primary causes of work-related disability in industrialized countries, CDDD is now classified among the “diseases of civilization”, steadily increasing in prevalence in association with ageing populations and instigating enormous health and economic costs globally [1,2]. Various conservative and surgical treatments have been proposed to preserve/restore nerve function, as well as relieve pressure and any ensuing sensory loss, pain, or tingling associated with CDDD. In particular, anterior cervical decompression and fusion (ACDF) surgery is widely performed as the gold standard treatment of CDDD with well-established results in terms of safety and efficacy [3]. However, long-term clinical studies on patients post ACDF indicate possible increased stiffness at the instrumented level, which is frequently associated with significant degenerative changes at the adjacent spinal levels, or so-called adjacent segment disease (ASD) [4]. ASD can be determined by the evaluation of the disc height loss, variation in sagittal segmental motion and lordosis angle, as well as the segmental degeneration index which indirectly measures disc hydration via an MRI imaging technique [5]. Indeed, radiographic and clinical adjacent level pathologies at prevalence rates of 92% and 19%, respectively, have been reported in patients post ACDF, including 7% of these patients who required revision surgery [6]. Pseudoarthrosis and intersegmental immobility have also been indicated subsequent to ACDF, emphasizing the critical need for considering these challenging outcomes a priori during surgical planning [7].

To decrease the risk of ASD and intersegmental immobility, total disc arthroplasty, or artificial disc replacement, has increasingly gained popularity in the last decade or so. Several types of artificial discs were patented based on metal-on-metal or metal-on-plastic designs, and few were successfully approved by the Food and Drug Administration (FDA) for the treatment of degenerative disc disease in the US and beyond [8,9]. Among these, the Bryan® cervical artificial disc (Medtronic, Memphis, TN, USA), classified within the metal-on-plastic design group, has demonstrated satisfactory clinical results [10,11], while the Prestige LP® cervical disc prosthesis (Medtronic, Memphis, TN, USA), considered as part of the newer generation of metal-on-metal devices, is known for allowing relatively simpler implantation techniques [11]. Despite some shortcomings, including endplate subsidence, hyper mobility, and clinical complications, cervical disc arthroplasty is today considered as a viable alternative to ACDF [9]. On the other hand, its superiority over traditional ACDF remains uncertain, especially considering that surgical outcomes may significantly vary depending on the particular patient's physiological and pathological status.

Numerous *in-vitro* experimental [12,13] and clinical studies [14,15] have been conducted over the years to evaluate the biomechanical response of artificial discs. Most of these have consistently demonstrated that the range of motion (ROM) at the surgical level of the spine is significantly greater with artificial disc implants as compared to ACDF, which is the core advantage of disc arthroplasty and may result in a favourable decrease of the risk of ASD [[16], [17], [18]]. Despite the valuable results obtained from these studies, relevant information on the detailed internal biomechanical response of the instrumented spine remains elusive.

In the past couple of decades, finite element (FE) modeling has emerged as a key cost- and time-effective tool for non-invasive biomechanical assessment in multiple clinical applications, not only minimizing the need for elaborate experimental studies, but also allowing investigators to address what if scenarios with minimal resources [19]. Numerous FE models were developed to study the biomechanics of the cervical spine with respect to different surgical treatments and implantation options [19,20]. However, most available studies considered static loading scenarios, and only very few considered the poroelastic time-dependent response of the cervical spine [21]. Investigating the biomechanical response of the cervical spine during dynamic loading and assessing the impact of time-dependent damping characteristics (i.e., the shock absorption mechanism) under cyclic loading are valuable in many applications, including spinal surgeries. The evaluation of the biomechanical long-term performance of different arthroplasty devices, for example, can reveal respective variations in the ROM, intradiscal pressure (IDP) and facet joint forces (FJFs), largely informing prognosis and treatment options. On the other hand, most existing relevant FE studies in literature for the biomechanical assessment of healthy, diseased and treated cervical spines are constrained to only one standard geometry, and, therefore, the effect of anatomical parameters on biomechanical responses for different patients is typically neglected [19,22,23]. Geometrically patient-specific FE models have the capability to overcome this limitation and provide more reliable results through considering personalized anatomical features. Meanwhile, considering the time-dependent response of the cervical spine, which is essential for the evaluation of the fluid–solid interaction of the IVDs, could have major impact on the model's prediction of adjacent segment biomechanics. Investigating the variation of ROM, IDP, FJFs, disc height loss, fluid loss, and fiber strain and stress may bridge the current gaps in clinical studies towards shedding more light on the risk of ASD post-surgery. Hence, This study aimed to comparatively investigate the biomechanics of the cervical spine, consequent to ACDF and arthroplasty with different devices (Bryan® and Prestige LP® artificial discs), subject to cyclic loading using a validated geometrically patient-specific poroelastic FE modeling approach.

## 2 Materials and methods

### 2.1 Development of the pre-operative geometrically patient-specific finite element models

**Fig. 1:**
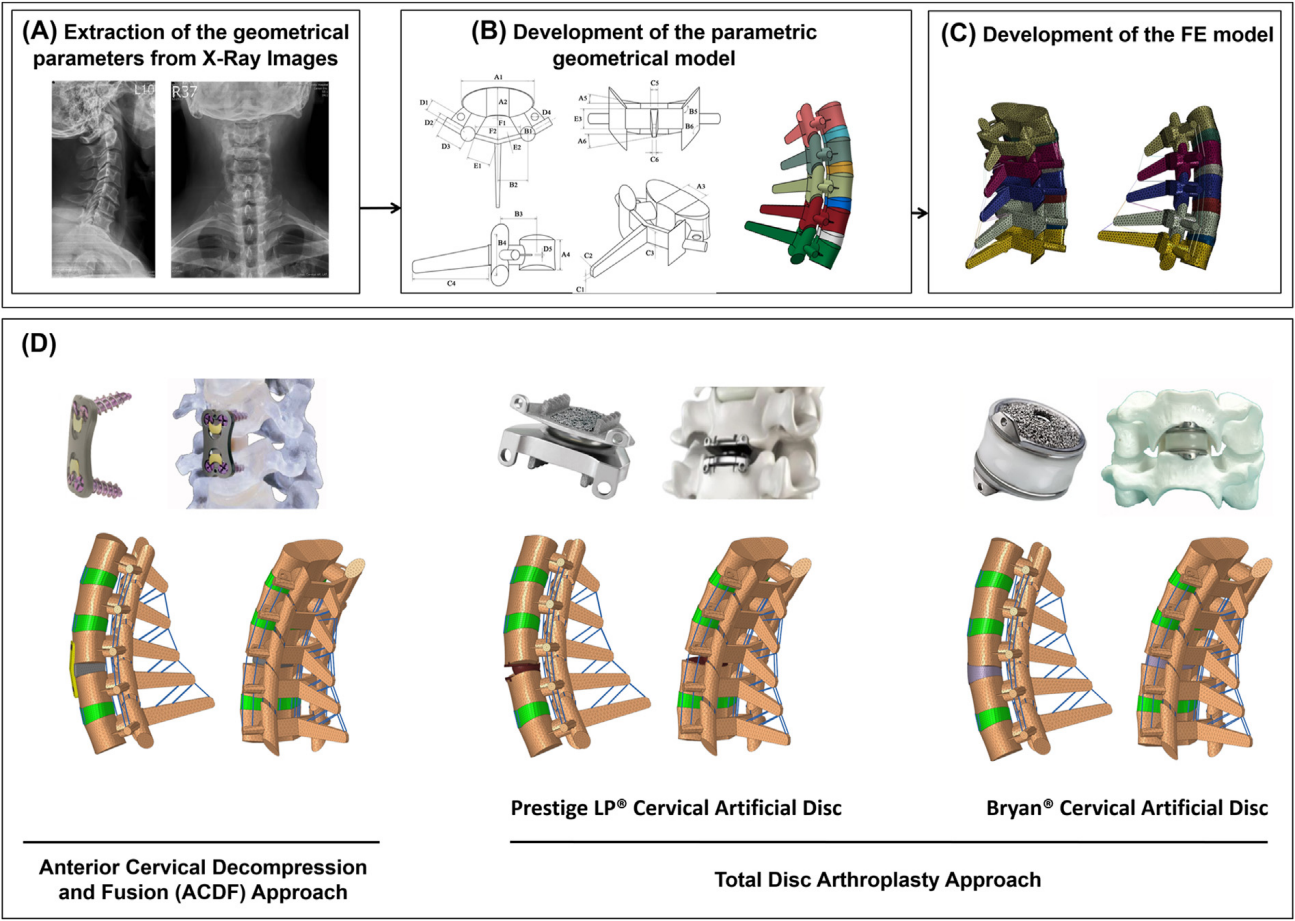
(A–C) The procedure outlining the patient-specific poroelastic FE modeling of the lower cervical spine and (D) Postoperative models including the anterior cervical decompression and fusion (ACDF), Prestige and Bryan total disc arthroplasty approaches.

The initial geometry of the lower cervical spine (C3–C7) was extracted from lateral and anterior-posterior (AP) X-ray images of 10 patients (6 males, 4 females; 46.8 ​± ​7.2 years old, 165.1 ​± ​7.3 ​cm, and 74.3 ​± ​7.1 ​kg) from the data bank compiled in our previous study [24,25]. A signed informed consent had been obtained from all participants prior to their enrolment in the study, and the protocol was approved by the institutional ethics review committee. The selected patients had single level symptomatic degenerative cervical disc disease and were eligible candidates for both ACDF and artificial disc arthroplasty surgical approaches at the C5–C6 level. The eligibility of patient selection was assessed and confirmed by an expert neurosurgeon. The images were imported to MIMICS (Materialise, Leuven, Belgium) for measurement of the selected parameters required for geometrical model development. A total of 28 parameters were marked on each vertebra on the lateral side, and AP images in the neutral position were used to extract the vertebral geometric parameters (Fig. 1B). Two extra parameters (i.e. the anterior height of the intervertebral disc (IVD) and the lordosis angle) were used to assemble five vertebrae and develop the final geometry [24]. Geometry of the IVD was built to fill the gap between the inferior surface of the upper vertebra and the superior surface of the lower vertebra (Fig. 1B). On both sides, the endplates were constructed between the vertebral bodies and IVDs using shell elements. The articular facet joint surfaces were approximated in our model by a plane in which the orientation was defined by two card angles (Fig. 1B). The geometry of the lower cervical spine (C3–C7) was automatically generated using the measured values from Lateral and AP X-ray images based on our previously validated algorithm (Fig. 1A–C) [24]. Subsequently, the geometrically patient-specific models for 10 patients were exported to Hypermesh (Hyperworks 12.0, Altair, USA) and the FE models were developed using ABAQUS (SIMULIA, Providence, RI, USA). The FE models included 5 typical lower cervical vertebrae, 4 IVDs, 4 pairs of facet joints (FJs), and 6 different ligaments (i.e., anterior longitudinal ligament (ALL), posterior longitudinal ligament (PLL), ligamentum flavum (LF), capsular ligament (CL), interspinous ligament (ISL) and Supraspinous ligament (SSL)). To mimic the anatomical structure of the IVDs, a composite material consisting of physiologically based simulated nucleus pulposus (NP) and AF regions reinforced by collagen fibers was used. The IVD volume was divided into NP and AF ground substance using the partition technique with a proportion of 56% NP and 44% AF, respectively. The drained solid phase for both the NP and AF was modeled based on the nonlinear Mooney–Rivlin hyperelastic model [26] (Table 1). The theory of poroelasticity was adopted for simulating the IVDs, endplates and vertebral bodies, and hence the mechanical structures of these tissues were simulated as interconnected pores saturated with fluid. The governing equations for saturated porous media are based on the equilibrium of the solid and fluid phases. Permeability characteristics were assumed dependent on void ratio variations (Table 1) [27] as follows:

(1) $$k=k_{0}{\left[\frac{e\left(1+e_{0}\right)}{e_{0}\left(1+e\right)}\right]}^{2}exp\left[M\left(\frac{1+e}{1+e_{0}}-1\right)\right]$$

where *k*_*0*_ is the initial permeability and *e* is the void ratio.

(2) $$e=\frac{\emptyset_{f}}{1-\emptyset_{f}}$$

where Ø_f_ is the porosity of the tissue. Swelling in the IVDs was simulated using a constant boundary pore pressure constraint of 0.25 ​MPa imposed on all the external surfaces. To mimic the anatomical collagen fibers structure and orientation, rebar elements were embedded in the ground substance matrix of the AF in 6 distinct concentric layers, and were arranged in an alternating crisscross manner with 25-degree orientation [28]. The endplates were constructed between the vertebral bodies and IVDs using shell elements. Surface-to surface tie contact conditions were used to constrain equal degrees of freedom (i.e., equal translational and rotational motions) for attached surfaces between the vertebral bodies, endplates, and IVDs. A gap distance of 0.3 ​mm was selected to simulate the articulation of the facet joints, and surface to surface contact in both tangential and normal directions was included in the model. An exponential over closure pressure [29] was employed to simulate normal contact rule, in addition to a tangential frictionless property. The ligaments were considered as nonlinear truss elements which can be activated only in tension [28]. Mechanical properties of other components were assumed as linear elastic based on the available data in literature (Table 1). This study used a total of 52,723 elements with 0.6 ​mm global element edge length to simulate the FE models based on a meshing sensitivity study.

**Table 1:** Material properties of the patient-specific finite element model.

Component		Mechanical Properties				References
Cortical Bone		E_xx_ ​= ​11300 ​MPa, G_xy_ ​= ​3800 ​MPa, υ_xy_ ​= ​0.484				[21,26]
		E_yy_ ​= ​11300 ​MPa, G_yz_ ​= ​5400 ​MPa, υ_yz_ ​= ​0.203				
		E_zz_ ​= ​22000 ​MPa, G_xz_ ​= ​5400 ​MPa, υ_xz_ ​= ​0.203 *k*_*0*_ ​= ​1 ​× ​10^−20^ (m^4^/Ns), e ​= ​0.02				
Cancellous Bone		E_xx_ ​= ​140 ​MPa, G_xy_ ​= ​48.3 ​MPa, υ_xy_ ​= ​0.45				[21,26]
		E_yy_ ​= ​140 ​MPa, G_yz_ ​= ​48.3 ​MPa, υ_yz_ ​= ​0.315				
		E_zz_ ​= ​200 ​MPa, G_xz_ ​= ​48.3 ​MPa, υ_xz_ ​= ​0.315 *k*_*0*_ ​= ​1 ​× ​10^−13^ (m^4^/Ns), e ​= ​0.4				
Endplate		E ​= ​5 ​MPa, ν ​= ​0.4, *k*_*0*_ ​= ​4 ​× ​10^−15^ (m^4^/Ns), e ​= ​4				[21]
Annulus Fibrosus Ground		Poro-Hyperelastic (Mooney-Rivilin)				[21]
		C1 ​= ​0.56, C2 ​= ​0.14, υ ​= ​0.45, *k*_*0*_ ​= ​1.82 ​× ​10^−16^ (m^4^/Ns), e ​= ​2.45				
Nucleus Pulposus		Poro-Hyperelastic (Mooney-Rivilin)				[21]
		C1 ​= ​0.12, C2 ​= ​0.09, υ ​= ​0.4999, *k*_*0*_ ​= ​1.82 ​× ​10^−16^ (m^4^/Ns), e ​= ​5.67				
Disc Fibers		Rebar elements, E ​= ​500 ​MPa, υ ​= ​0.3				
Cervical Plate/Screws		E ​= ​110000 ​MPa, ν ​= ​0.3				[28]
PEEK Cervical Interbody Cage		E ​= ​3500 ​MPa, ν ​= ​0.3				[23,36]
Bryan®		Outer Titanium Surfaces: E ​= ​110000 ​MPa, ν ​= ​0.3				[23,36]
		Nucleus: E ​= ​30 ​MPa, ν ​= ​0.3				
		Sheath: E ​= ​30 ​MPa, ν ​= ​0.3				
Prestige LP®		Titanium: E ​= ​110000 ​MPa, ν ​= ​0.3				[23,36]
Ligaments		Nonlinear Tension-only Truss				[24,28]
Cross-section Area of the Ligaments (mm^2^)						
Level	ALL		PLL	LF	CL	ISL
C3–C5	11.1		11.3	46.0	42.2	13.0
C5–C7	12.1		14.7	48.9	49.5	13.4

To confirm the validity of the calculated results based on the pre-operative (Pre-op) FE models, a pure moment of 1 ​N ​m was applied in different directions (i.e., flexion, extension, right and left lateral bending, right and left axial rotation). The moment was applied to the center of the superior C3 vertebra, while all the degrees of freedom for the inferior C7 vertebra were set to zero to represent a fixed surface point. The pre-op results of the intersegmental ROM were compared to data from experimental studies in the literature [30], while the IDP in neutral position subject to a compressive follower load (100 ​N), was compared to *in-vitro* experimental data [31].

### 2.2 Development of the post-operative geometrically patient-specific finite element models

Post-operative (post-op) models were reconstructed for all 10 patients for comparing the biomechanical impact of ACDF with different types of disc arthroplasty on the lower cervical spine. The lower cervical FE model was modified at the C5–C6 level to simulate 3 post-op models for each patient (a total of 30 post-op models), where the ALL ligament was removed, and discectomy was considered for all post-op models. The models for different implants were developed using simplified geometries based on the measured parameters. To match the proper heights of the implanted devices, standard values were selected from the manufacturers’ product catalogues. An anterior cervical polyetheretherketone (PEEK) interbody cage, filled with bone graft, was inserted at the C5–C6 level for the ACDF model, while 2 types of artificial discs (i.e, Bryan® and Prestige LP® artificial discs) were inserted at the C5–C6 level for simulating cervical disc arthroplasty (Fig. 1B). The superior and inferior surfaces of the PEEK interbody cage and artificial discs were attached to the respective vertebral bodies using tied contact to simulate complete osteointegration of the devices with the bone to ensure no relative motion (i.e., no translational or rotational motions) between the implant and vertebral endplates. The contact between the metal and metal surfaces of the Prestige LP® artificial disc was modeled as a surface to surface contact with a coefficient of friction of 0.1 [23].

Following a 30 ​min preconditioning resting period under a constant compressive load of 46N to replicate the weight of the head and remedy the overstated swelling in the beginning of the analysis, a cyclic compression load with an amplitude of 100N and frequency of 0.5 ​Hz (i.e. in the form of F ​= ​50 ​+ ​50 cos(π(t-1))), was applied to the post-op FE models. The cyclic axial compressive loading was simulated based on the follower load technique using connector elements for 11000 cycles [32]. Rotational movements, including flexion, extension, right and left lateral bending, right and left axial rotation were superimposed using 1 ​N ​m moment (to mimic the physiological loading of the spine [30]) before and after cyclic loading. The aforementioned moments were applied to the centroid of the superior surface of C3, and Dirichlet boundary conditions were considered at the inferior surface of C7. The rotational moments were linearly applied and removed during 4 ​s (i.e., 2s for loading and 2s for unloading), and only one motion was evaluated in each simulation to eliminate the errors regarding the loading combinations (i.e., 6 individual simulations for each model). Biomechanical responses, including intersegmental ROMs (i.e., the rotation angle of the IVDs), disc height loss (i.e., reduction of IVD heights at mid-point), disc fluid loss (i.e., the average variation of the fluid flow in the disc based on calculated void ratios), axial effective stress in the AF, and collagen fiber strain, were analyzed before and after cyclic loading under the same loading and boundary conditions. The non-parametric Friedman with Nemenyi *post hoc* tests were used to evaluate the statistical differences of the results, where *p* values less than 0.05 were considered as significant. This statistical test, which is a non-parametric statistical comparative test, was used here towards a robust comparative investigation based on within-subject differences.

## 3 Results

**Fig. 2:**
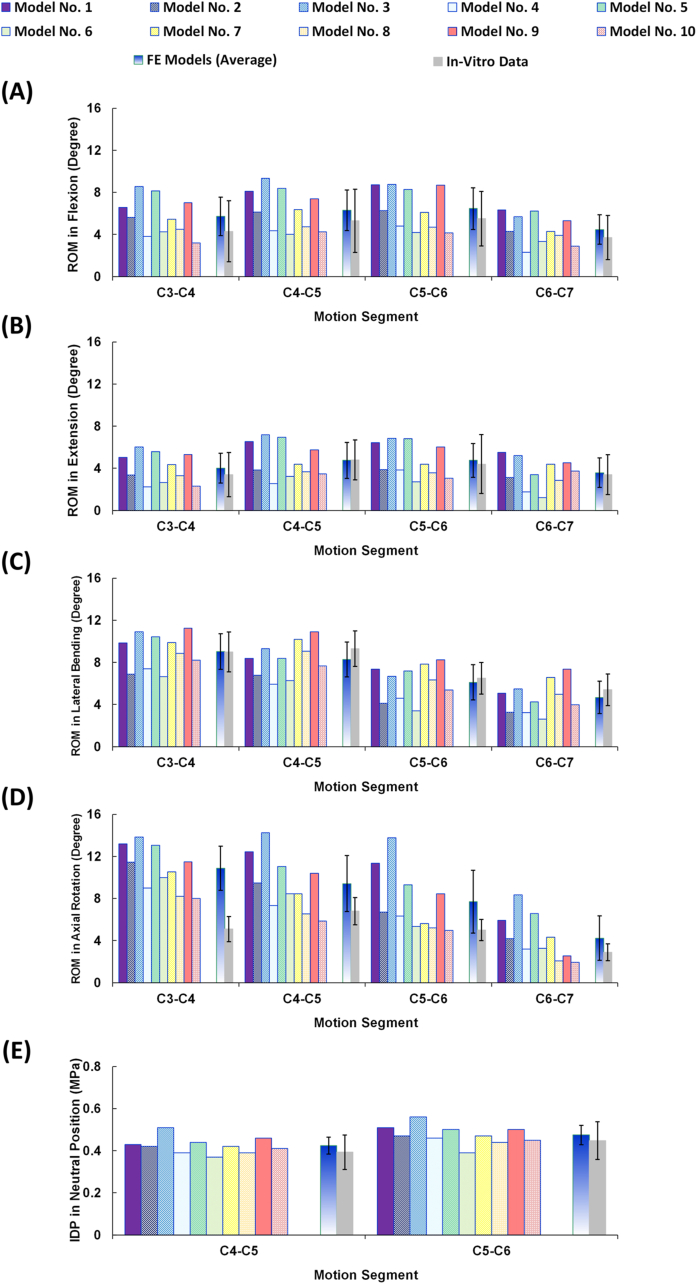
Intersegmental ROMs for pre-op FE models (N ​= ​10) compared to *in-vitro* experiments [30] in (A) flexion, (B) extension, (C) lateral bending, and (D) axial rotation (E) Intradiscal pressure (IDP) for pre-op FE models (N ​= ​10) in neutral position subject to 100N compressive follower load compared to *in-vitro* experiments [31].

The FE models were successfully reconstructed using patient-specific updated algorithms, and the accuracy of all models was tested by evaluating the mesh independency. The calculated intersegmental ROMs for C3 to C7 and the IDP values for C4–C5 and C5–C6 were found to be well within the reported range of available *in-vitro* data from literature [30,31] (Fig. 2). The resulting average ROMs for the entire lower cervical model (C3–C7) were 22.94 (±7.14), 17.12 (±6.12), 28.10 (±6.50), and 32.24 (±9.82) degrees, for flexion, extension, lateral bending, and axial rotation, respectively (Fig. 2), in alignment with *in-vitro* experimental data from literature [30]. The average IDP values for neutral posture were 0.424 (±0.041) and 0.475 (±0.046) MPa for C4–C5 and C5–C6, respectively, which is also within the reported range of previous *in-vitro* work [31] (Fig. 2).

**Fig. 3:**
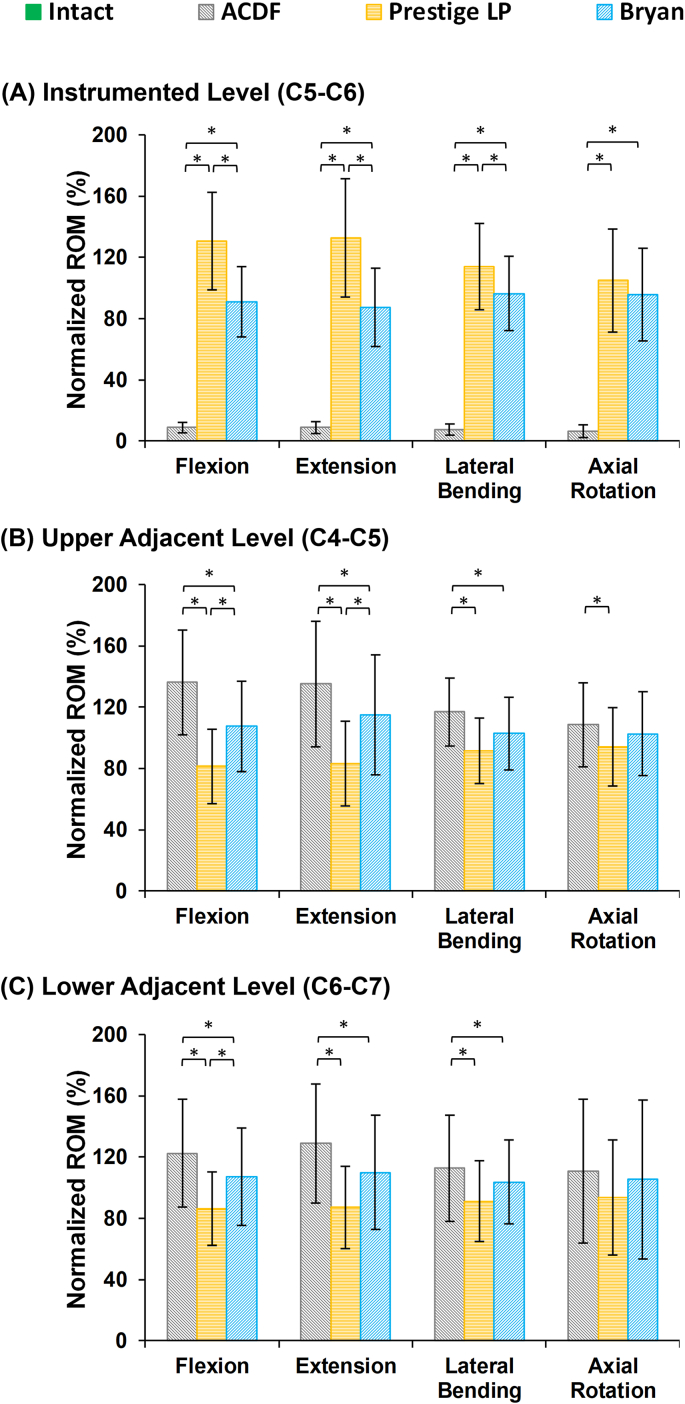
Normalized intersegmental ROMs for lower cervical FE models following different surgeries at the (A) instrumented level (C5–C6), (B) upper adjacent level (C4–C5), and (C) lower adjacent level (C6–C7) for different movements. The ROMs were normalized relative to the calculated values in the intact model.

The results of the post-op FE models revealed that the ROMs at the instrumented levels (i.e., C5–C6) significantly decreased for the ACDF model as compared to intact, at an average of 0.57° in flexion, 0.42° in extension, 0.46° in lateral bending, and 0.51° in axial rotation (Fig. 3A). Conversely, the ROMs at the instrumented level were higher for the artificial disc models in comparison to the ACDF model (Fig. 3A). Moreover, the ROMs for the Prestige LP® artificial disc model were significantly higher as compared to the Bryan® model (Fig. 3A). In terms of the ROMs at the adjacent levels (i.e., C4–C5 and C6–C7), they significantly increased in the ACDF model as compared to intact and to both types of artificial disc models (i.e, Bryan® and Prestige LP® artificial discs), particularly during flexion and extension (Fig. 3B and C), respectively. However, the observed variations were not statistically significant during axial rotation movements (Fig. 3).

**Fig. 4:**
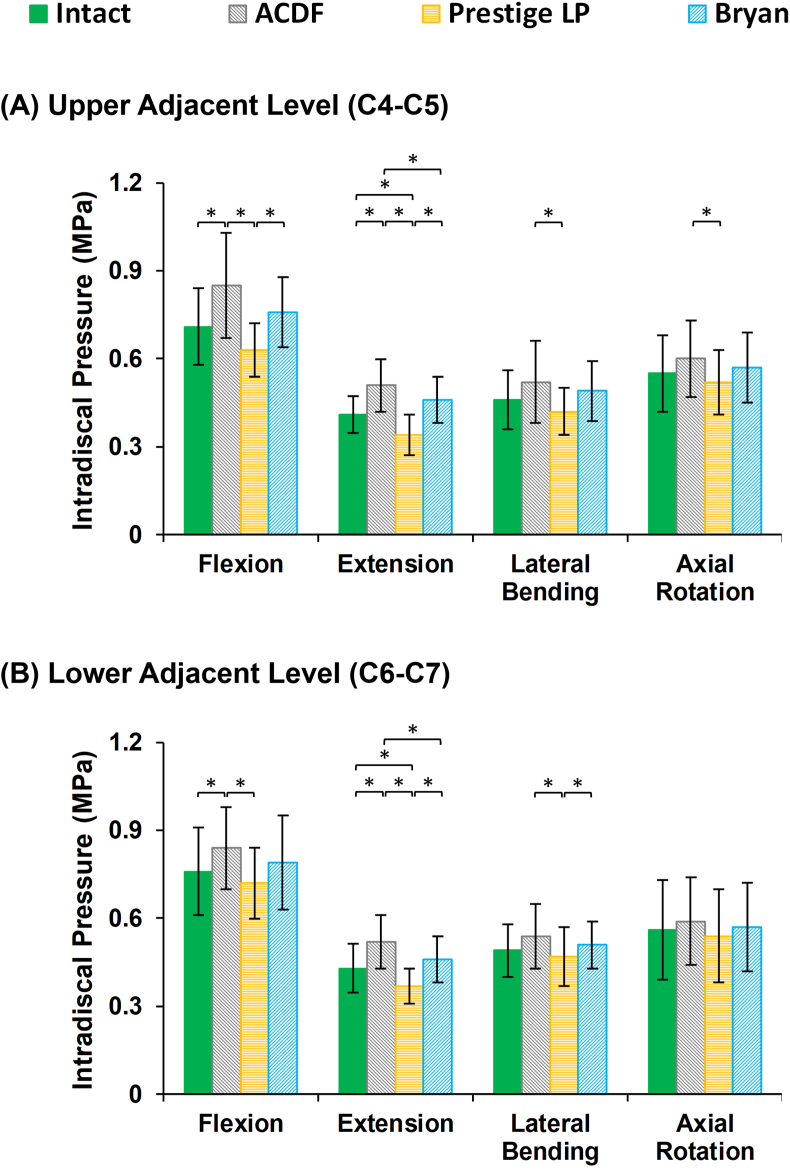
Intradiscal pressure (IDP) values for lower cervical FE models following different surgeries at the (A) upper adjacent level (C4–C5), and (B) lower adjacent level (C6–C7) for different movements.

**Fig. 5:**
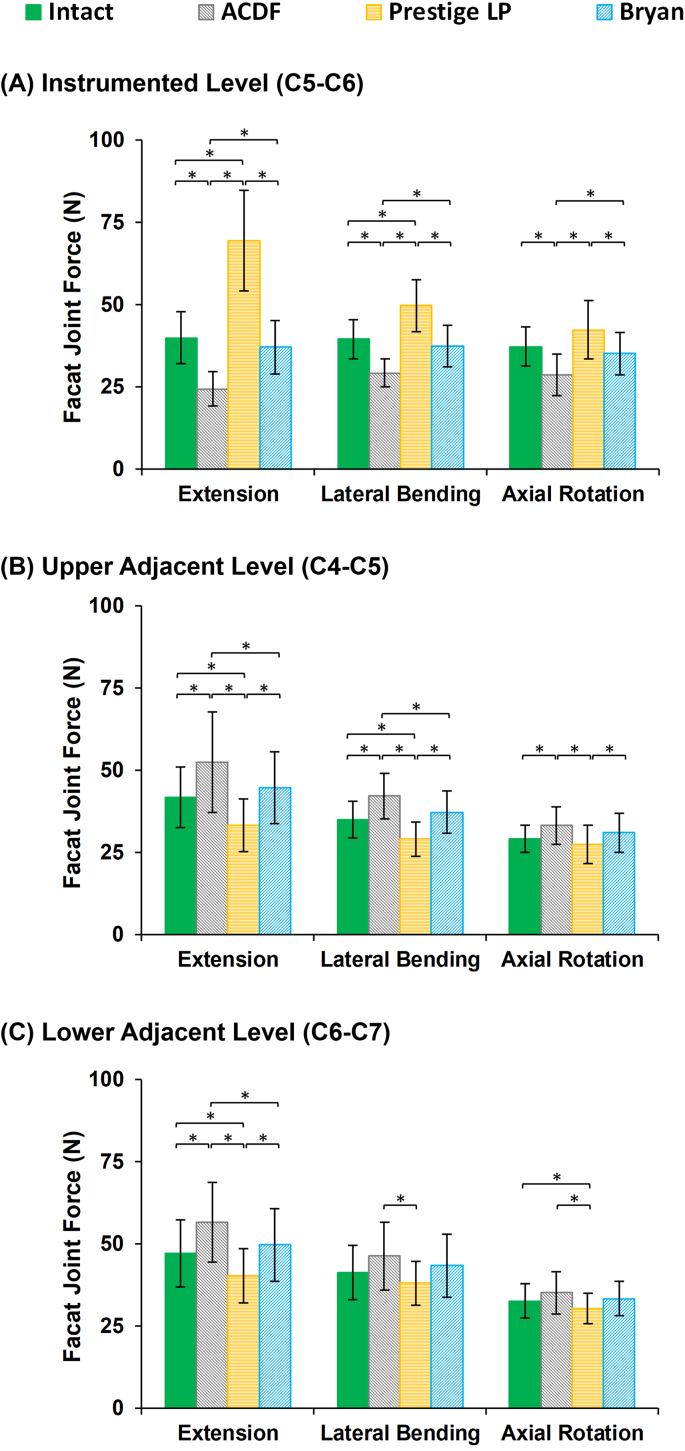
Facet joint forces (FJF) values for lower cervical FE models after different surgeries at the (A) instrumented level (C5–C6), (B) upper adjacent level (C4–C5), and (C) lower adjacent level (C6–C7) for different movements.

Similar trends were seen in the IDP (Fig. 4) and FJF values (Fig. 5) at the surgical levels. Both the IDPs and FJFs values increased at the adjacent levels in the ACDF models as compared to the intact models during sagittal plane motion (Fig. 4, Fig. 5). The increased FJF at the instrumented level (C5–C6) was remarkable in the Prestige LP® artificial disc models (Fig. 5). On the other hand, the variations in the values of the IDPs were not significant on average for different models during lateral bending and axial rotation motions.

**Fig. 6:**
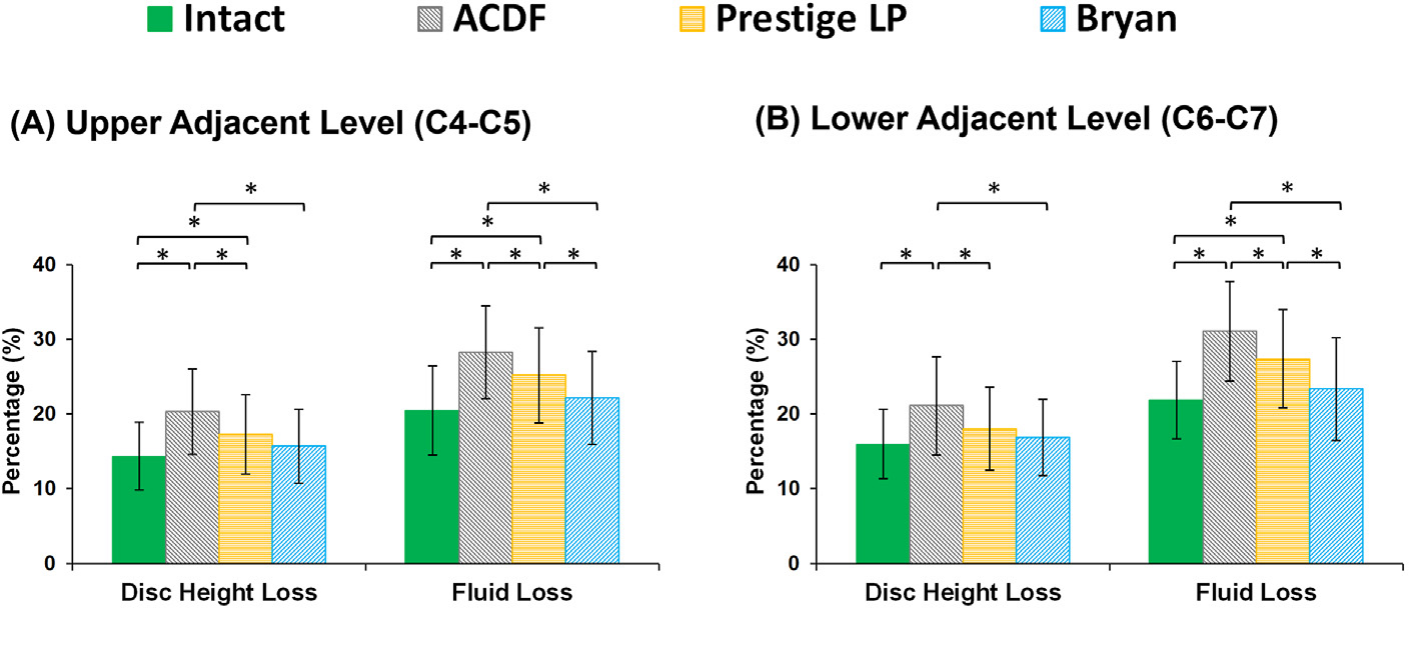
Variation in disc height loss and fluid loss for post-op lower cervical FE models at the (A) upper adjacent level (C4–C5) and (B) lower adjacent level (C6–C7).

**Fig. 7:**
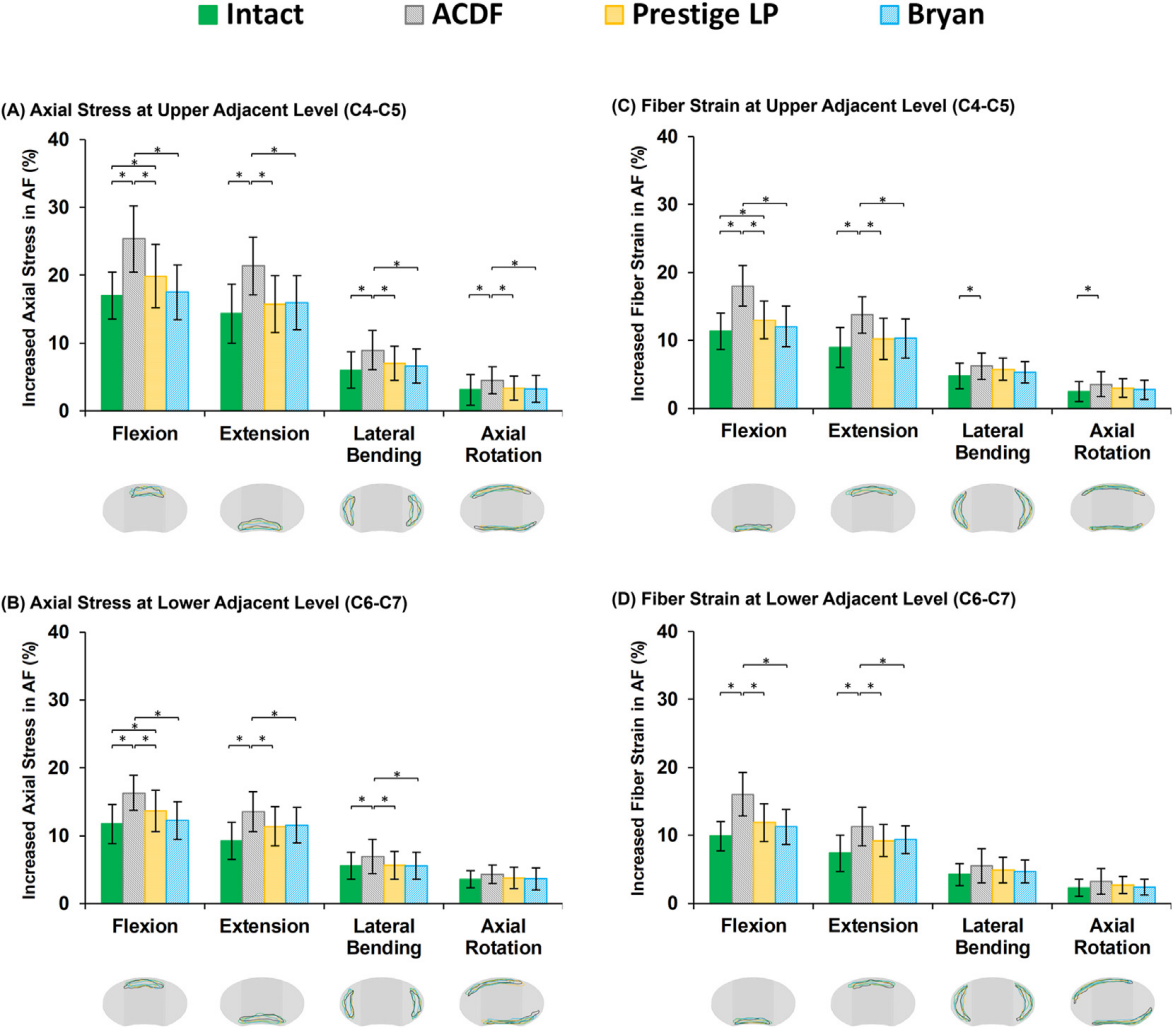
Increased axial stress in AF for post-op lower cervical FE models at the (A) upper adjacent level (C4–C5) and (B) lower adjacent level (C6–C7) for different movements. Increased fiber strain in AF for post-op lower cervical FE models at the (C) upper adjacent level (C4–C5) and (D) lower adjacent level (C6–C7) for different movements. ∗The regions with the highest calculated stress and fiber strain for different models were presented in schematic views of the IVDs for different rotational movements.

Disc height loss was on average 14.36%, 15.27% and 15.98% at the C4–C5, C5–C6, and C6–C7, respectively for the pre-op models, after 11000 cycles. The average fluid loss during cyclic loading was 20.45%, 19.36% and 21.89% at the C4–C5, C5–C6, and C6–C7, respectively, for the pre-op models. For the ACDF models, both disc height loss and fluid loss at the adjacent levels were significantly increased as compared to the pre-op models (Fig. 6). By contrast, the amount of disc height loss and fluid loss were lower at the adjacent IVDs for the artificial discs (Fig. 6), as compared to the ACDF models. The lowest values in disc height and fluid exchange were observed in the Bryan® artificial disc models (Fig. 6). Similar trends were observed for the AF axial stress and collagen fiber strain, which were significantly higher for the ACDF models (Fig. 7). The altered values for increased stress and fiber strain at the adjacent levels were minimal during lateral bending and axial rotations (Fig. 7).

## 4 Discussion

ACDF continues to be the gold standard treatment for CDDD due to its well-established safety and efficacy records [3]. On the other hand, reported results in over 90% of patients who undergo ACDF indicate decreased ROM and increased risk of ASD, and hence warrant looking into alternative treatment options. Artificial disc arthroplasty was largely introduced to restore the flexible function of surgically removed intervertebral discs and minimize the incidence of ASD [[16], [17], [18],^33^]. Indeed, studies show that cervical disc arthroplasty can provide desirable motion patterns at the instrumented segment(s) and help recover the physiological load sharing after surgery. Nonetheless, many clinicians agree that the optimal treatment option for CDDD should be patient-specific and planned according to the patient's particular pathological condition, lifestyle, and demographics, as well as the surgeon's experience, skill set and available technologies. Comparative biomechanical analyses of the lower cervical spine, following different treatment strategies based on patient-specific FE models, allow for informed personalized treatment and potentially improved short- and long-term outcomes. In this study, a geometrically parametric FE modelling approach was developed providing patient-specific pre- and post-op simulations for 10 different patients to compare and contrast the biomechanical responses of the cervical spine subsequent to different types of common surgeries (A total of 10 pre-op and 30 post-op FE models).

The results for the 10 patient-specific pre-op models were generally in alignment with *in-vitro* experimental data adopted from literature [30,31]. However, it is common in FE analysis for the model responses not to completely match the observed experimental data. The only major variations observed in this study were associated with the ROM in axial rotation at the C3–C4 level which was greater for the FE models as compared to the *in-vitro* data (Fig. 2D). This is most likely due to geometry simplification adopted from literature, where flat instead of convex/concave surfaces were utilized to simulate the articular facet joints. Nonetheless, the aforementioned variation was only observed in axial rotation and only at the upper level of the model. The results of the current parametric modeling approach were overall in good agreement with our previous FE geometrically-accurate model. This suggests that the simplified geometry did not significantly affect the ROM, IDP, nor the fiber strain, as confirmed by our previous geometrically-accurate model [24]. However, the individual patient-specific FE models reflected big variations in the intersegmental motion patterns, confirming the significant effect of patient-specific geometrical parameters in the global response of the cervical spine. Up to date, most relevant computational modelling efforts in literature use one particular geometry for the cervical spine, hence limiting the ability for assessing the effect of anatomical parameters on different treatment approaches [24]. Simulations integrating the influence of a patient's anatomical and anthropometric parameters (vertebral dimensions, disc heights, cervical curvature, etc.), along with proper biomechanical analyses, allow for better quantitative evaluation of different anterior cervical instrumentation, shedding light on short- and long-term surgical outcomes and biomechanical implications.

A PEEK cervical interbody cage was used to simulate the ACDF models based on the surgical techniques employed in our data bank, although both PEEK and porous metal interbody cages (i.e., Titanium/Tantalum interbody cages) are commonly utilized in clinics. We also considered osteointegration of the devices with the bone in this study to ensure no relative motion, neither translational nor rotational, in simulating the ACDF models. Hence, changing the material properties of the interbody cage only alters the stress distribution of the cage and the attached vertebral surfaces at the fused level with no significant effect on the comparative analyses. Post-op spinal biomechanics associated with two different artificial disc devices, representing two typical cervical disc arthroplasty examples, were evaluated in this study. The Bryan® artificial disc mimics the natural anatomy and function of the physiological intervertebral disc with a polyurethane nucleus component fitted between two titanium alloy shells (Fig. 1). Each titanium shell has an outer porous coated surface to support bony ingrowth and long-term stability, while the polyurethane component simulates the physiological damping response of intervertebral disc. The Prestige LP® artificial disc, on the other hand, represents one of the most popular metal-on-metal ball-in-socket sliding articulation designs [34], and preserves disc mobility by allowing rotational with translational motion while maintaining proper alignment (curvature and height) of the natural disc. As previously detailed, artificial disc arthroplasty devices are employed to minimize the shortcomings of rigid ACDF systems, mainly in terms of the ROM at the instrumented level and associated risk of ASD. However, these non-fusion techniques are costly, and cannot be used for patients who still require fusion surgery due to cervical spine instability. Both ACDF and artificial disc arthroplasty surgical approaches are today commonly practiced in clinics, and hence quantitative, non-invasive biomechanical analyses to compare and contrast these techniques are merited.

To assess the outcome of anterior surgical techniques on adjacent segment biomechanics, it is essential to consider the time-dependent response of the cervical spine, which was one of the main contributions of the current study. ASD, a long-term phenomenon, requires evaluating the responses of the cervical spine under cyclic loading, in addition to considering the poroelastic theory for calculation of the fluid–solid interactions in the IVDs, endplates, and vertebrae, which have mostly been neglected in previous works. In addition, we used a constant boundary pore pressure constraint imposed on all the external surfaces to mimic the swelling phenomenon in the IVDs. Although this is one the simplest models for simulation of soft tissue swelling, it is considered as an acceptable approximation when focusing on global spinal kinematics [35]. The authors have successfully used this technique in our published lumbar spine models [36]. In this study, we used a cyclic loading scenario of 11000 cycles with frequency of 0.5 ​Hz. This was based on the estimation of the number of steps during walking for 19 ​km in line with literature [37]. The trends of our results were averagely stabilized after around 7300 cycles for all models. Although this loading scenario may be different from a typical long-term post-operation loading history (which is not possible to be simulated numerically), it provides a practical framework for estimating the variation in the biomechanical responses before and after cyclic loading. The results obtained here can be generalized for the prediction of long-term responses of the cervical spine post-surgery. Therefore, one of the main contributions of the current study is predicting realistic responses by considering the endurance of the IVD during repetitive loading conditions. The disc height loss and fluid loss were compared between pre-op and post-op models, providing two quantitative clinical markers/indicators for the prediction of the risk of ASD [38]. The alteration in motion patterns in different directions was also compared before and after cyclic loading to evaluate the resulting stress and strain variations in the adjacent discs, which provides yet another potential clinical indicator of the risk of adjacent disc degeneration.

Our findings based on post-op FE simulations revealed that the ROMs at the instrumented surgical level (C5–C6), as compared to the pre-op models, significantly decreased post ACDF, but were higher on average for the Prestige LP® artificial disc. Conversely, the reverse happened for the adjacent discs, where the ROMs significantly increased post ACDF, but were lower on average for the Prestige LP® artificial disc, as compared to the pre-op models. The ROMs at both instrumented and adjacent levels were closest to the pre-op models for the Bryan® artificial disc (i.e. slightly lower at the instrumented level and slightly higher at the adjacent levels). Our results also indicated that although the artificial discs minimized abnormal alterations at the adjacent levels by reducing the ROM, IDP, and FJF, the Prestige LP® artificial disc dramatically increased the FJFs at the instrumented level, as compared to pre-op models. This phenomenon can be attributed to the hypermobility of the instrumented level in the Prestige LP® artificial disc implantation. In previous clinical studies, similar results were observed, where the ROM significantly increased at the instrumented level post Prestige LP® implantation [11,39]. The hypermobility condition, which may be considered as a negative factor, can shift the load sharing through the posterior parts leading to an increase in the FJF. Importantly, the standard deviations observed in this study reflected scattered values for different patients with different anthropometric and geometric parameters, which confirms the impact of personalized patient-specificity as compared to the one size fits all traditional approach. For example, this study shows that the ROMs at the adjacent levels were lower for the Bryan® disc as compared to the Prestige LP® for 3 of the patients, while the reverse trend was observed for the remaining 7 patients.

Comparing the results of the post-op to the pre-op intact models revealed that although the percentage of disc height loss and fluid loss were generally uniform at different spinal levels, they were significantly altered in the post-op models, especially for the ACDF models. This suggests that increasing the rigidity at the instrumented level allows transferring the load sharing through the adjacent segments, which may result in increasing the disc height loss and alter the fluid–solid interaction of the disc. Previous clinical studies observed significant disc height loss in adjacent segments, which may signal the initial stage of adjacent disc degeneration for patients who undergo anterior cervical fusion surgery [38]. The current study demonstrated that cervical disc arthroplasty has better clinical outcomes, as compared to traditional anterior fusion, despite the biomechanical alterations in comparison with pre-op models. Greater fluid loss during cyclic loading was observed in association with the ACDF approach. This may have minimized the contribution of the fluid phase in the bulk stiffness of the IVD matrix, hence leading to higher stress and strain in the solid phase. Recognizing that disc height loss and fluid loss constitute relevant clinical measures of the spine's damping performance, suggests that artificial disc arthroplasty mimics the natural physiological biomechanical response of the spine. However, the faithfulness of these biomechanical patterns depends on the implant's design, as evidenced by the superior performance of the Bryan® implant. Similar trends were observed in terms of the stress and fiber strain in the AF region for the different surgical approaches. Notably, the stress in the AF region and the collagen fiber strain were significantly increased at the adjacent levels post ACDF both in flexion and extension, as compared to pre-op and artificial disc post-op models.

Certain limitations in the current study should be deliberated here. The first one is related to the development of the FE models based on simple symmetric shapes (e.g., circles, rectangles, and ellipses) using X-Ray images. On the other hand, our previous comparative analyses using both parametric and geometrically-accurate models with the same geometry, indicated similar trends in the global response (i.e., ROM, IDP, fiber strain), which confirms the capability of this modeling approach [24]. The added advantages of the simplified parametric technique adopted here in terms of time and computational cost, as well as, ease of updating patient-specific data, justified the simplifications and rendered the approach much more clinically applicable. The second limitation lies in the use of similar mechanical properties in the FE models of the various patients. This unavoidable constraint is due to the fact that up to date, there is no guideline in literature on how to discriminate/extract patient-specific material properties from X-Ray images. Although this limitation may affect the attained results, the proposed simplification can be tolerated since the objective of this study was to comparatively analyse the biomechanical impact of different surgical approaches. Future work can be enhanced with patient-specific mechanical properties if available. Thirdly, only the passive responses of muscle forces were considered in this study based on the follower load methodology, and the effects of muscle forces were neglected in our calculations. Although this is a common simplification in related studies [19], a supplementary dynamic algorithm would enhance this work and can be coupled with the current FE methodology for future work.

## 5 Conclusion

This study revealed greater motion pattern, higher values of stress and strain in the AF region, and increased disc height and fluid loss at the adjacent levels post ACDF, as compared with pre-op results and both types of artificial disc arthroplasty, suggesting a higher risk for adjacent disc degeneration. The artificial disc implants studied here (Bryan® and Prestige LP®) not only preserved the motion at the instrumented level, but also sustained the pre-op ROM at the adjacent levels. The advantages of using artificial discs, as compared to ACDF, include decreasing the IDP and FJF at the adjacent levels, particularly during flexion and extension. However, the FJF values at the instrumented level was greater after Prestige LP® artificial disc implantation which happens based on the hypermobility of the instrumented level and may be considered as a negative factor. Furthermore, the results demonstrate that the Bryan® implant, in particular, could be more effective for sustaining the natural poroelastic characteristics of adjacent IVDs. This study shows that geometrically patient-specific FE modeling can effectively be used as a surgical planning tool in clinical settings to compare among different treatment options towards choosing the proper solution for optimal clinical outcomes.

## Author contribution

Category 1

Conception and design of study: Kinda Khalaf, Mohammad Nikkhoo.

acquisition of data: Kinda Khalaf, Mohammad Nikkhoo.

analysis and/or interpretation of data: Mohammad Nikkhoo.

Category 2

Drafting the manuscript: Kinda Khalaf and Mohammad Nikkhoo.

revising the manuscript critically for important intellectual content: Kinda Khalaf.

Category 3

Approval of the version of the manuscript to be published (the names of all authors must be listed):

## Funding

The authors acknowledge the scientific funding support from the Khalifa University and Health Engineering Innovation Center (HEIC).

## Declaration of competing interest

The authors declare that the research was conducted in the absence of any commercial or financial relationships that could be construed as a potential conflict of interest.
